# Enhancing Adherence to Continuous Positive Airway Pressure Therapy in Patients With Obstructive Sleep Apnea Using Augmented Reality: Protocol for a Randomized Controlled Trial

**DOI:** 10.2196/69757

**Published:** 2025-05-06

**Authors:** Ming-Che Chen, Yen-Chin Chen, Cheng-Yu Lin

**Affiliations:** 1 Department of Electronic Engineering Southern Taiwan University of Science and Technology Tainan Taiwan; 2 Department of Nursing, College of Medicine National Sun Yat-sen University Kaohsiung City Taiwan; 3 Department of Nursing, College of Medicine National Cheng Kung University Tainan Taiwan; 4 Department of Otolaryngology College of Medicine, National Cheng Kung University Hospital National Cheng Kung University Tainan City Taiwan; 5 Sleep Medicine Center National Cheng Kung University Hospital, College of Medicine, National Cheng Kung University Tainan Taiwan

**Keywords:** obstructive sleep apnea, continuous positive airway pressure, augmented reality, adherence, feasibility

## Abstract

**Background:**

Continuous positive airway pressure (CPAP) therapy is the gold standard treatment for treating obstructive sleep apnea (OSA). However, patient adherence to CPAP therapy remains a critical challenge, with many individuals finding it difficult to maintain consistent use due to discomfort, lack of understanding, or perceived inconvenience. Augmented reality (AR) offers a novel approach to overcoming these barriers by providing interactive real-time guidance to users, potentially enhancing adherence rates.

**Objective:**

This clinical trial aims to examine the feasibility of AR implementation and the effect of AR on improving CPAP adherence in patients with OSA.

**Methods:**

A randomized controlled trial with a parallel assignment will be conducted. Participants will be randomly assigned to either an experimental group receiving AR-guided CPAP therapy or a control group receiving standard care. The study will span 6 months, with assessments at baseline (T0), and with follow-ups at 1 month (T1), 3 months (T2), and 6 months (T3) post intervention. The primary outcome measure is CPAP adherence, defined as using the CPAP device for more than 70% of sleep time, with a minimum of 4 hours per night. Secondary outcomes will evaluate the common adverse effects associated with CPAP therapy, device usability, and time required for CPAP machine use education.

**Results:**

This study is funded by the Ministry of Science and Technology, Taiwan (August 2023 to July 2026) and was registered in August 2024 (ClinicalTrials.gov NCT06520592). Participant recruitment is scheduled to begin in April 2025, and by September 2025, we expect to have enrolled 40 participants (50% of the target sample of 80). Preliminary analyses of CPAP adherence at 1 month and usability data are currently underway. Final data collection is anticipated to be completed by December 2025, with results expected to be published by Fall 2026.

**Conclusions:**

Anticipated findings suggest that AR-guided CPAP therapy may significantly enhance patient adherence by improving mask fitting and providing effective, interactive education. If validated, this innovative approach could pave the way for more personalized technology-driven interventions in OSA management and other chronic conditions requiring long-term therapy adherence.

**Trial Registration:**

ClinicalTrials.gov NCT06520592; https://clinicaltrials.gov/study/NCT06520592

**International Registered Report Identifier (IRRID):**

PRR1-10.2196/69757

## Introduction

Obstructive sleep apnea (OSA) is a prevalent sleep disorder characterized by repetitive episodes of upper airway obstruction during sleep, which results in low oxygen saturation or frequent awakenings [1]. It has been associated with significant health risks, including cardiovascular diseases [2], cognitive impairment [3], and decreased quality of life [4]. Continuous positive airway pressure (CPAP) therapy is a strongly recommended treatment for OSA according to the clinical practice guidelines of the American Academy of Sleep Medicine. It effectively mitigates the associated risks by maintaining airway patency during sleep [5]. However, adherence to CPAP therapy remains a significant challenge, as many patients struggle to use the device consistently due to physical discomfort [6] and lack of proper guidance or without follow-up care [7].

Guiding patients in disease-related knowledge through educational materials to change their adherence to medical treatment is a commonly used method in clinical practice [8]. However, the effectiveness of guiding middle-aged and older individuals through printed educational materials is often limited. Kim and Oh [9] conducted qualitative interviews with 16 frontline nurses in South Korea and attempted to examine the situation of older adults in receiving health education and health literacy through their perspective. The study found that older adults struggle to understand educational content due to declines in physical and cognitive functions.

The Information-Motivation-Behavioral Skills model suggests that patients’ adherence behavior is driven by their level of information, motivation, and behavioral skills [10]. Augmented reality (AR) applications have the potential to significantly enhance patient adherence to therapeutic regimens through engagement and understanding [9,11]. AR applications can provide personalized real-time information in an engaging format. A systematic review and meta-analysis, which included 11 articles, found that AR can be a tool in physiotherapy to improve older adults’ balance, especially in patients with Parkinson disease [11]. In addition, AR can enhance motivation through interactive features, such as progress tracking and gamification, which make the therapy experience more rewarding [12]. Ideally, by guiding patients in the correct use of their devices, AR can also enhance the behavioral skills necessary for successful adherence.

Previous studies have explored various interventions to improve CPAP adherence, including standardized care procedures [13], cognitive behavioral therapy [14], troubleshooting [15], and telemonitoring [16]. However, the integration of AR technology into CPAP management is a novel approach. Limited research has been conducted on the application of AR in this context, making this study one of the first to investigate its potential impact on OSA management. By addressing the gap in the literature, this study aims to contribute to the growing body of knowledge on innovative strategies to enhance adherence to CPAP therapy.

This study hypothesizes that AR-guided CPAP therapy can enhance patient engagement and understanding of CPAP therapy among patients with OSA, and patients receiving AR-guided CPAP therapy will show higher adherence rates compared to those receiving standard care.

## Methods

### Study Design

This parallel group randomized controlled trial is designed to evaluate the effectiveness of AR-guided CPAP therapy on patient adherence among individuals diagnosed with OSA. Participants will be randomly assigned to either the experimental group (AR-guided therapy) or the control group (standard care) by the blocking sample method. Aside from the AR component, both groups will receive identical standard care. An assistant researcher (Ms Ng) generated the random allocation sequence, case managers enrolled participants with OSA, and an assistant researcher (Ms Jhong) assigned participants to interventions. In this study, we will blind assessors and investigators. The study will follow participants over 6 months, with data collection points at baseline (T0), and follow-ups at 1 month (T1), 3 months (T2), and 6 months (T3) post intervention.

### Participants

Patients with OSA will be randomly assigned to either the experimental group or the standard care group.

#### Inclusion Criteria

Our study will enroll adults 20 years or older diagnosed with moderate to severe OSA (Apnea-Hypopnea Index ≥15) confirmed by polysomnography who are CPAP therapy naive at the time of enrollment.

#### Exclusion Criteria

We will exclude those with previous use of positive pressure ventilators, a diagnosis of central sleep apnea, a presence of uncontrolled acute mental illness, or a terminal illness.

#### Sample Size

The sample size was determined based on a power analysis conducted for the CPAP adherence rate. Effect sizes were estimated to be 0.32 based on our previous findings, which indicated that 32.25% of CPAP-related side effects were associated with the CPAP interface [17]. A power of 0.85 was used to detect a significant difference at *P*=.05 (2-sided). Given the highly variable dropout rate for CPAP therapy, we aimed to accommodate a 20% dropout rate, which necessitated recruiting 80 participants, with 40 participants in each group.

#### Experimental Group Receiving AR-Guided CPAP Therapy: Proposed System

**Figure 1:**
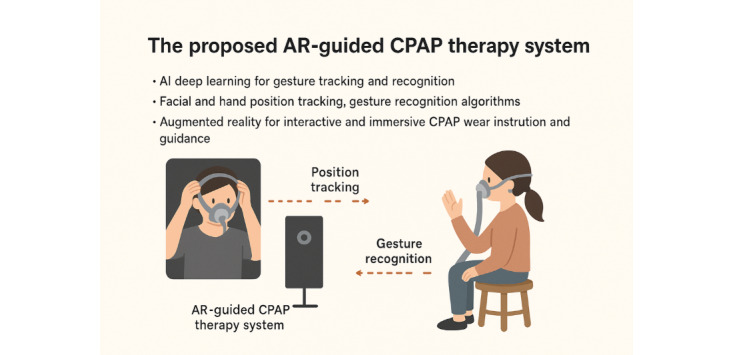
Proposed system architecture.

Figure 1 illustrates the proposed system architecture, consisting of a camera module, an Edge-AI computing module, and a display module. The camera module captures the user’s facial and hand images, while the Edge-AI computing module processes these images to detect relevant features and perform calculations to ensure a proper nasal mask fit and verify the accuracy of the wearing procedure. The display module presents the measurement results and offers real-time feedback on the recommended nasal mask size, following the mask sizing guidelines provided by ResMed [18].

#### Standard Care Group

Participants assigned to the standard care group will have their mask size visually checked by a case manager, who will also teach them how to use the CPAP machine.

### Primary Outcomes

#### Objective Measure: Adherence to CPAP Therapy

CPAP adherence will be monitored daily using AirView, a cloud-based remote monitoring system. The research team will explain the study objectives to participants and obtain their written consent to authorize ResMed to grant access to their CPAP cloud data. This approval will enable the research team to retrieve and analyze adherence data with company authorization.

Data from CPAP devices (eg, AirSense 10 AutoSet) will be collected at T1, T2, and T3, tracking the percentage of nights meeting adherence criteria. Adherence refers to the patient’s adherence to CPAP therapy, which means using CPAP for more than 70% of the total sleep time during the night, with a minimum duration of 4 hours. The calculation method involves dividing the number of days with CPAP use exceeding 4 hours by the total number of days and multiplying by 100 to obtain the average percentage of CPAP use [19].

#### Subjective Measure: Adherence to CPAP Therapy Questionnaire

We developed a self-assessed CPAP adherence and comfort levels questionnaire to allow participants to track their daily adherence and comfort levels, providing a comprehensive overview of their subjective CPAP experience.

- Adherence is defined as using CPAP for at least 70% of total sleep time per night, with a minimum duration of 4 hours.
- Comfort levels are self-rated on a 0 to 10 scale, where 0 is extremely uncomfortable and 10 is extremely comfortable.
- If participants experience discomfort, they will be asked to document the specific reasons contributing to their discomfort (Multimedia Appendix 1).

This structured daily assessment enables a detailed evaluation of patient experiences, helping to identify potential barriers to CPAP use and providing valuable insights into factors influencing adherence and comfort over time.

### Secondary Outcomes

#### Common Adverse Effects Associated With CPAP

Common discomforts of using CPAP were collected and categorized based on symptoms identified by Australian scholars Ghadiri and Grunstein [6].

#### Device Usability

At the end of the study, participants will fill out a system usability scale (Multimedia Appendix 2) to evaluate system usability. This scale is a questionnaire assessing the usability of emerging technologies or systems, consisting of 10 items scored from 0 to 4 (from strongly disagree to strongly agree). The overall score is then multiplied by 2.5, with a maximum score of 100. According to Sauro [20], based on nearly 500 studies, a score of 68 is used as the benchmark for the system usability assessment: if the score is greater than 68, it indicates that the system’s usability is above 50%; if it is less than 68, the opposite is true.

#### Accuracy of Estimated Nasal Size

The AR system’s recommended nasal size compared to the case manager’s estimation shows an agreement rate approaching 95% at T0.

#### Time Required for CPAP Machine Use Education

We will calculate the time during the initial education session, hands-on training, patient questions and answers, and troubleshooting guidance at T1.

### Covariates

#### Demographic Data

We will collect participants’ gender, height, weight, waist circumference, blood pressure values, education level, presence of other comorbidities (eg, hypertension, diabetes, cardiovascular diseases, chronic obstructive pulmonary disease, asthma, cancer), smoking habits (none, once a week, once a month, once a year), drinking habits (none, once a week, once a month, once a year), exercise habits (none, once a week, once a month, once a year), history of being diagnosed with mental health disorders, napping habits, and the approximate duration of naps.

#### Clinical Indicators

The Apnea-Hypopnea Index, average blood oxygen saturation, and minimum blood oxygen saturation will be measured through polysomnography and a CPAP machine.

### Procedure

**Figure 2:**
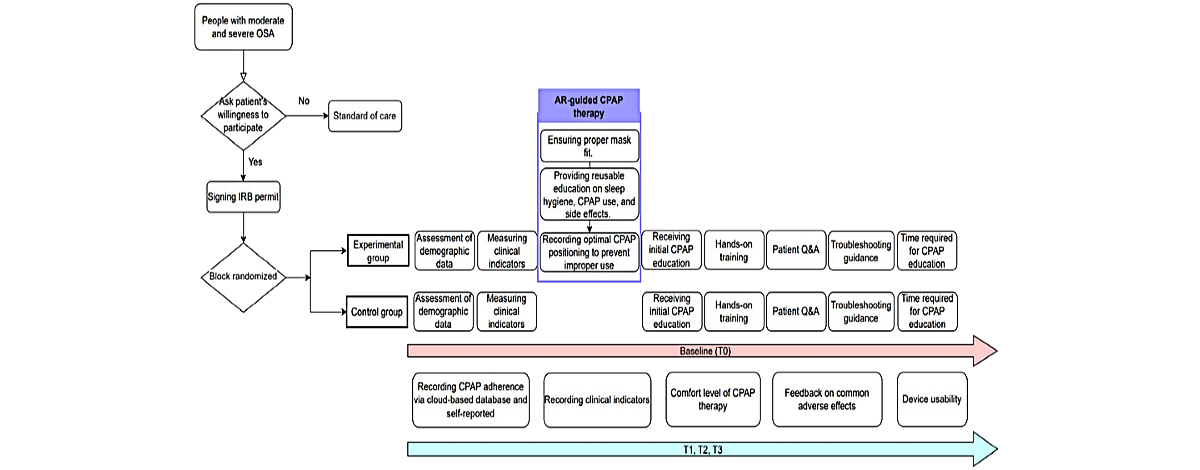
AR-guided CPAP therapy procedure followed in both the experimental group and the standard care group during the study. Data collection points were at baseline (T0), and follow-ups at 1 month (T1), 3 months (T2), and 6 months (T3) post intervention. AR: augmented reality; CPAP: continuously positive airway pressure; IRB: institutional review board; OSA: obstructive sleep apnea; Q&A: questions and answers.

Participants diagnosed with moderate to severe OSA through polysomnography will be assessed for their willingness to participate in the study by the principal investigator. Patients who agree to undergo CPAP therapy and sign the institutional review board permit will be referred to the clinical trial. A research assistant will then contact potential participants via telephone to reconfirm their willingness to enroll. Upon confirmation, the research assistant will assist in scheduling the clinical trial and coordinating regular follow-ups at T1, T2, and T3 to assess CPAP adherence and evaluate treatment-related adverse effects (Figure 2).

### Ethical Considerations

#### Human Subject Ethics Review

This study was approved by the Institutional Review Board of National Cheng Kung University on March 1, 2023 (approval A-ER-111-531). The study adheres to all institutional guidelines and ethical principles outlined in the Declaration of Helsinki.

#### Informed Consent

All participants will provide written informed consent before enrollment. They will receive detailed explanations regarding the study objectives, procedures, and potential risks and benefits. Participants will have the right to withdraw from the study at any time without consequences.

#### Privacy and Confidentiality

All collected data will be anonymized and deidentified to protect participant privacy. Identifiable information will be securely stored and accessed only by authorized research personnel. Data will be reported in an aggregate format, ensuring that individual participants cannot be identified.

#### Compensation Details

Participants will receive financial compensation (US $6) for T2 and T3 participation. Besides that, they will be provided with comprehensive CPAP education and follow-up support as part of the study. No additional costs will be incurred by participants for their involvement.

#### No Identifiable Features of Research Participants

We confirm that no identifiable information of individual participants or users is present in any images included in the manuscript or supplementary materials.

### Data Analysis

To evaluate the effectiveness of AR-guided CPAP therapy on telemonitored adherence rates and self-reported CPAP adherence, a mixed-effects model for repeated measures will be used. This model accounts for missing data and enables comparisons of adherence rates between the two groups at T0, T1, T2, and T3. Correlation analyses will be conducted between objective CPAP adherence and the subjective adherence assessment. Data analysis was performed using SPSS version 22.0 (IBM Corp).

## Results

This randomized controlled trial is funded by the Ministry of Science and Technology Council, Taiwan, from August 2023 to July 2026. The trial was registered in August 2024 (ClinicalTrials.gov NCT06520592). Participant recruitment is scheduled to commence in April 2025, with follow-up assessments planned at 1 month (T1), 3 months (T2), and 6 months (T3) post intervention.

We plan to enroll 40 participants by September 2025, representing 50% of the targeted sample size of 80. Preliminary analyses of baseline adherence and usability data are underway. The trial remains on schedule, with the final data collection anticipated in December 2025 and results expected to be published by Fall 2026.

## Discussion

### Summary of Principal Findings

This prospective observational study is anticipated to yield valuable insights into the effectiveness of AR-guided CPAP therapy in improving adherence among patients with OSA. Specifically, the study focuses on facilitating the selection of appropriately fitting mask sizes, providing hands-on training on proper mask use, and offering troubleshooting support. If the hypothesis is validated, AR technology could emerge as a powerful tool to enhance patient engagement and address machine interface barriers to CPAP adherence, such as discomfort, leakage, and lack of motivation. The study’s results could have broader implications for the management of OSA and other chronic conditions that require long-term adherence to therapeutic regimens.

### Comparison to Prior Work

Previous studies have explored telemonitoring and cognitive behavioral therapy to improve CPAP adherence, but research integrating AR technology into CPAP management remains limited. By addressing this gap, our study contributes to the growing evidence on technology-assisted interventions for chronic disease management.

### Strengths and Limitations

A strength of this study is the comprehensive assessment of adherence, incorporating both objective adherence tracking and subjective self-reported adherence evaluation. This multidimensional approach enhances the study’s ability to identify barriers to CPAP adherence and potential intervention strategies to improve long-term compliance. Additionally, this study has a randomized controlled design, which enhances the validity and reliability of the findings. Another strength is that the integration of AR technology allows for real-time patient education and support, which has the potential to improve CPAP adherence through enhanced engagement and interactive learning.

However, some potential limitations should be considered. First, generalizability may be limited due to the specific population studied. Since the study is conducted in a single region with a particular demographic profile, findings may not be fully applicable to other populations with different cultural, socioeconomic, or health care structures. Second, the novelty of AR technology may present implementation challenges, particularly among older adults or patients with low technology literacy, potentially limiting the accessibility and effectiveness of the intervention. Lastly, variations in AR device compatibility across different CPAP models could pose practical barriers to widespread adoption.

### Future Directions

To address these limitations, future research should explore multicenter studies across diverse populations and investigate strategies to enhance AR accessibility for less tech-savvy users, such as simplified interfaces or additional training modules.

### Dissemination Plan

The results of this study will be disseminated through peer-reviewed publications, conference presentations, and clinical workshops. Additionally, findings may inform the development of AR-based educational modules for sleep medicine practitioners.

### Conclusions

This study is expected to provide valuable insights into the role of AR-guided CPAP therapy in improving adherence among patients with OSA. By integrating real-time interactive guidance, the intervention aims to enhance patient engagement, improve mask fitting, and address adherence barriers such as discomfort and lack of motivation. While findings from this study may support the use of technology-driven interventions in CPAP management, further research will be needed to explore long-term effectiveness, broader applicability across different populations, and strategies to optimize accessibility for patients with lower technology literacy. If validated, this study could inform future clinical practice by integrating AR-based patient education and support tools into standard CPAP care, potentially improving adherence and long-term treatment outcomes for patients with OSA.

## Appendix

Multimedia Appendix 1 Subjective Questionnaire: CPAP Adherence and Comfort Assessment.

Multimedia Appendix 2 System Usability Scale.

## Abbreviations

AR: augmented reality
CPAP: continuous positive airway pressure
OSA: obstructive sleep apnea

## Data Availability

The data that support the findings of this study are available from the corresponding author upon reasonable request.
