# Supplementary material for: Enhancing Adherence to Continuous Positive Airway Pressure Therapy in Patients With Obstructive Sleep Apnea Using Augmented Reality: Protocol for a Randomized Controlled Trial
Source: JMIR Res Protoc. 2025 May 6;14:e69757. doi: 10.2196/69757 (PMC12093067; doi:10.2196/69757)
Supplement: Multimedia Appendix 1 [file resprot_v14i1e69757_app1.docx]

Supplementary Table 1. Subjective Questionnaire: CPAP Adherence and Comfort Assessment

1. CPAP Adherence and self-assessed comfort levels (Example: one-month tracking)

| Day | A | C | Reasons | Day | A | C | Reasons | Day | A | C | Reasons | Day | A | C | Reasons |
| --- | --- | --- | --- | --- | --- | --- | --- | --- | --- | --- | --- | --- | --- | --- | --- |
| 1 |  |  |  | 8 |  |  |  | 15 |  |  |  | 22 |  |  |  |
| 2 |  |  |  | 9 |  |  |  | 16 |  |  |  | 23 |  |  |  |
| 3 |  |  |  | 10 |  |  |  | 17 |  |  |  | 24 |  |  |  |
| 4 |  |  |  | 11 |  |  |  | 18 |  |  |  | 25 |  |  |  |
| 5 |  |  |  | 12 |  |  |  | 19 |  |  |  | 26 |  |  |  |
| 6 |  |  |  | 13 |  |  |  | 20 |  |  |  | 27 |  |  |  |
| 7 |  |  |  | 14 |  |  |  | 21 |  |  |  | 28 |  |  |  |

Adherence (A) means using CPAP for more than 70% of the total sleep time during the night, with a minimum duration of 4 hours.

Self-assessed Comfort Levels (C): Participants will rate their comfort level while using CPAP on a 0 to 10 scale, from 0 = Extremely Uncomfortable to 10 = Extremely Comfortable.

Reasons for discomfort or non-adherence
