# Supplementary material for: Enhancing Adherence to Continuous Positive Airway Pressure Therapy in Patients With Obstructive Sleep Apnea Using Augmented Reality: Protocol for a Randomized Controlled Trial
Source: JMIR Res Protoc. 2025 May 6;14:e69757. doi: 10.2196/69757 (PMC12093067; doi:10.2196/69757)
Supplement: Multimedia Appendix 2 [file resprot_v14i1e69757_app2.docx]

Supplementary Table 2. System Usability Scale

| Number | Items |
| --- | --- |
| 1 | I think that I would like to use this platform frequently. |
| 2 | I found the platform unnecessarily complex. |
| 3 | I thought the platform was easy to use. |
| 4 | I think that I would need the support of a technical person to be able to use this system. |
| 5 | I found the various functions in this platform were well integrated. |
| 6 | I thought there was too much inconsistency in this platform. |
| 7 | I would imagine that most people would learn to use this platform very quickly. |
| 8 | I found the platform very awkward to use. |
| 9 | I felt very confident using the platform. |
| 10 | I needed to learn a lot of things before I could get going with this platform. |
